# The effect of exposure to high altitude and low oxygen on intestinal microbial communities in mice

**DOI:** 10.1371/journal.pone.0203701

**Published:** 2018-09-12

**Authors:** Wei Zhang, Lefei Jiao, Ruixin Liu, Yu Zhang, Qiaorong Ji, Huan Zhang, Xiang Gao, Yan Ma, Hai Ning Shi

**Affiliations:** 1 Research Center for High Altitude Medicine, Medical College of Qinghai University, Xining, Qinghai, China; 2 Qinghai Key Laboratory of Science and Technology for High Altitude Medicine, Xining, China; 3 Mucosal Immunology and Biology Research Center, Massachusetts General Hospital and Harvard Medical School, Charlestown, Massachusetts, United States of America; 4 Weinan Central Hospital, Weinan, Shaanxi, China; Sichuan University, CHINA

## Abstract

This experiment was conducted to investigate the effect of exposure to high altitude and low oxygen on intestinal microbial communities using mice as an animal model. Fecal microbiota from mice housed in a control environment representing 2,200 meters (NC group) above sea level with 16% Oxygen and mice that were placed in a hypobaric chamber representing 5000 meters (HC group) above sea level with 11% Oxygen for 30 days, were analyzed by the HiSeq Illumina sequencing platform. The results showed a significant difference in beta diversity observed between the two groups, while no significant difference was observed in alpha diversity. Compared with the NC group, the relative abundance of class *Epsilonproteobacteria*, phlym *Actinobacteria*, class *Erysipelotrichia* and genus *Helicobacter* were significantly lower (*P*<0.05), while the relative abundance of genus *Alistipes* was increased in the HC group; Phenotypic analysis showed no significant difference in aerobic, anaerobic, facultatively anaerobic, potentially pathogenic, stress tolerant, mobile element, biofilms formation, gram negative and gram positive between HC group and NC group; Functional analysis results showed significant differences in 34 gene functional metabolic pathways (carbohydrate digestion and absorption, energy metabolism, arachidonic acid metabolism, flavonoid biosynthesis, RIG-I-like receptor signaling pathway, etc) between HC group and NC group. Together, these findings suggest that exposure to high altitude and low oxygen had the potential to change the intestinal microbial communities, which potentially may modulate metabolic processes in mice.

## Introduction

The Qinghai-Tibet Plateau is known as the "roof of the world." With an average elevation of 4,000 meters, the atmospheric pressure of oxygen is about 50% -60% of that at sea level [1, 2]. Hypoxia, therefore, is an extremely significant environmental factor that affects health and disease in both human as well as animal populations of this high-plateau area [3]. Residents of the Qinghai-Tibet Plateau represent some of the highest altitude populations on earth, and native Tibetans living on the Qinghai-Tibet plateau have gradually adapted to the special plateau environment and developed unique genetic predispositions, lifestyles, and dietary habits [4]. In recent years, with the growing interest in adventure travel and the increasing ease and affordability of air, rail, and road-based transportations, millions of people from plains areas come to the plateau for travel and work purposes. The number of the newcomers has increased every year [5, 6]. High altitude hypoxia is a tremendous challenge for people from plain areas. The reduction in barometric pressure and ambient oxygen levels trigger a series of pathophysiological consequences and lead to altitude illness [6]. Apart from neurological and pulmonary syndrome, many sojourners also experience gastrointestinal disorders such as anorexia, epigastric discomfort, flatus expulsion, dyspepsia, severe acidity, infectious diarrhea, etc [7, 8].

The gastrointestinal tract is colonized with an enormous community of microbes, which plays an important role in health and disease [9, 10]. Although the factors that shape the structure of intestinal microbiota remain to be fully appreciated, host genetic background as well as environmental factors (dietary), have been believed to play a key role in the modulation of the intestinal microbial communities [11, 12]. Information is now becoming available to indicate that the intestinal microbial community is also affected by geographical altitude. It has been shown that gut microbiota composition is different between people living in high-altitude areas and those living in low-attitude locations [4, 13]. However, apart from geographic origins, dietary habit is still recognized as a main factor affecting gut microbiota [14]. Recent studies have raised the question whether hypoxia at high altitude could modify GI microbial imprint and subsequently causes intestinal dysfunction. A few researchers have used bacterial culture technology or fluorescent in situ hybridization to investigate the change of intestinal microbiota in people who migrate or travel from low-altitude areas to high altitude places [15, 16]. However, due to technical limitations, both of the above technologies are not suitable to study comprehensive changes of microbiota; it is less clear to what extent intestinal microbial communities are affected by exposure to high altitude and low oxygenand how they are affected.

Therefore, the aim of this study was to investigate the effect of exposure to high altitude and low oxygen on intestinal microbial communities by HiSeq Illumina sequencing platform. In this current well-controlled investigation, fecal microbiota from mice housed at control environment—NC group, 2200 meters (582 mmHg, partial oxygen pressure (PO_2_) is 121.6 mmHg with 16% Oxygen), and mice that were placed in a hypobaric chamber exposed to high altitude of 5000 meters (HC group, 405 mmHg, PO_2_ is 84.7 mmHg, with 11% Oxygen) for 30 days were analyzed and compared. The atmospheric pressure at a given altitude was calculated based on published model (Portland State Aerospace Society, 2004, accessed 05032011).

Moreover, sequencing 16S ribosomal RNA from HiSeq Illumina sequencing platform reveals alterations to taxonomic groups and species composition, but it does not provide data on metabolic activity and function of the microbial communities. PICRUSt is an approach for predicting changes to microbial function which was likely associated with changes in OTU-abundance [17], while BugBase is a bioinformatic tool for analyzing wide-scale microbial phenotypes [18]. Hence, we also used PICRUSt and bugBase to predict functional and phenotypic changes in the microbiota of mice with exposure of low oxygen and high altitude.

## Materials and methods

### Mice

Six- to eight-week-old female Balb/c mice (Beijing Vital River Laboratory Animal Technology Co., Ltd., Beijing) were fed autoclaved food and water in individually ventilated cages (IVC), and maintained in a specific-pathogen-free facility at Medical College of Qinghai University. Animal care was provided in accordance with protocols approved by the Institutional Animal Care and Use Committee of Medical College of Qinghai University.

### Hypoxia treatment

Twelve female Balb/c mice were randomly divided into two groups (6 animals per group): low altitude group and high altitude group. For the low altitude group, mice were raised in animal facility 2200 m above sea level. For the high altitude group, mice were housed 5000 m above sea level in a controlled hypobaric chamber (DYC-3000, 8m×3m×3m, Guizhou Guizhou Fenglei Aviation Ordnance Co., Ltd, Guizhou, China) with IVC to keep exposure hypoxia air for 30 days.

### Blood sample collection

At the end of experiments (after hypoxia exposure for 30 days), mice were anesthetized by i. p. injection of 2.5% avertin solution and blood samples were collected through cardiac puncture [19]. Hematology and biochemistry samples were analyzed with Fully Automated Blood Cell analyzer (BM-800, Beijing).

### Fecal sample collection

Fresh fecal samples were collected and transferred in 2 mL sterile centrifuge tubes, and quickly transferred into a -80°C cryogenic freezer for cryopreservation.

### Fecal microbiome analysis

#### DNA extraction and PCR amplification

Total genome DNA from samples was extracted using CTAB method. DNA concentration and purity was monitored on 1% agarose gels. According to the concentration, DNA was diluted to 1ng/μL using sterile water. The V4 region of 16S rRNA gene (300–350 bp) was amplified using specific primer (515F : GTG CCA GCM GCC GCG GTAA; 806R : GGA CTA CHV GGG TWT CTA AT) with the barcode. All PCR reactions were carried out with Phusion® High-Fidelity PCR Master Mix (New England Biolabs).

#### PCR products quantification and qualification

PCR products were visualized on a 2% agarose gel and amplicons between 400–450 bp were chosen for further processing.

#### PCR products mixing and purification

PCR products were mixed in equidensity ratios. Then, mixed PCR products were purified with Qiagen Gel Extraction Kit (Qiagen, Germany).

#### Library preparation and sequencing

Sequencing libraries were generated using TruSeq® DNA PCR-Free Sample Preparation Kit (Illumina, USA) following manufacturer's recommendations and index codes were added. The library quality was assessed on the Qubit@ 2.0 Fluorometer (Thermo Scientific) and Agilent Bioanalyzer 2100 system. At last, the library was sequenced on an Illumina HiSeq 2500 platform and 250 bp paired-end reads were generated. Sequencing and analysis of the stool DNA were performed at Novogene Company (Beijing, China)

### Data analysis

Paired-end reads were assigned to samples based on their unique barcode and truncated by cutting off the barcode and primer sequence. Paired-end reads were merged using FLASH (V1.2.7, http://ccb.jhu.edu/software/FLASH/) [20].The high-quality, clean and effective tags were obtained through quality filtering [21, 22] and chimera removal [23]. Sequences analysis was performed by Uparse software (Uparse v7.0.1001, http://drive5.com/uparse/) [24]. Sequences with ≥97% similarity were assigned to the same OTUs. Representative sequence for each OTU was screened for further annotation. OTUs abundance information was normalized using a standard of sequence number corresponding to the sample with the least sequences. Subsequent analysis of alpha diversity and beta diversity were all performed based on this output normalized data. Alpha diversity was applied in analyzing complexity of species diversity for a sample. Among the Alpha diversity indices, Simpson, Shannon, Chao, ACE and Goods_coverage were calculated with QIIME (Version 1.7.0) while rarefaction curves was displayed with R software (Version 2.15.3). The microbial distribution in the samples was visualized using R package (Version 2.15.3) based on community composition information at taxonomic levels. Beta diversity (Non-Metric Multi-Dimensional Scaling, Anosim) was used as a comparative analysis of microbial communities in different samples. Non-Metric Multi-Dimensional Scaling (NMDS) and Anosim were analyzed using the R package vegan. The phylogenetic investigation of communities by reconstruction of unobserved states (PICRUSt) (Galaxy Version 1.0.0) was used to analyze the enrichment of functional genes in the fecal microbiota of each group. BugBase (http://github.com/danknights/bugbase) was used to calculate differences between both groups in terms of microbial phenotypes.

## Results

### Hematology and biochemistry results

Polycythemia is a common response to altitude hypoxia. In our study, we compare hematological and biochemical parameters between NC- and HC-treated mice. It was observed that HC group (at the 5000 meters) had significantly increased values of Hb and Hct of HC group than that detected in NC groupat the 2200  meters (as shown in Table 1). These results prove that the 30 day hypoxia exposure is effective.

**Table 1:** Changes of hematocrit (Hct) and hemoglobin (Hb) content in BABL/c mouse (n = 6).

Sample name	Hct (%)	Hb (g/L)
HC	66.26±0.89*	215.38±1.44*
NC	50.26±1.01	150.66±1.23

Mean ± SEM, n = 10.

* *P*<0.05. vs NC

### Characteristics of HiSeq sequencing results

**Fig 1:**
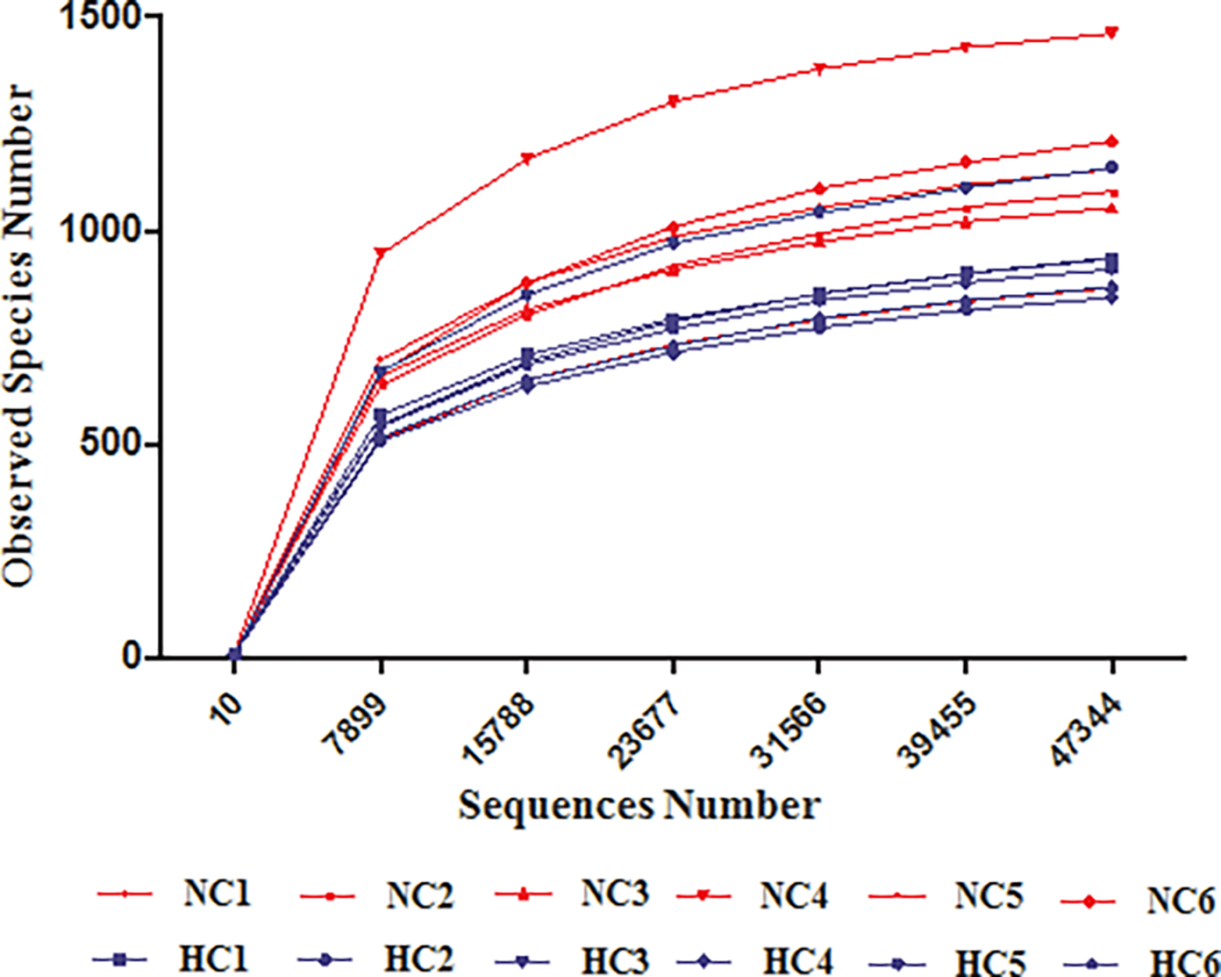
Rarefaction analysis. Rarefaction curves of OTUs clustered at 97% sequence identity across the sample. The sample labeled with NC1, NC2, NC3, NC4, NC5 and NC6 correspond to six replicates of NC group; HC1, HC2, HC3, HC4, HC5 and HC6 correspond to six replicates of HC group.

All of the polymerase chain reaction (PCR) products from 16S rRNA V4 region (515F-806R) of stool DNA from 12 animals (6 from a normal animal facility, 6 from high altitude of 5000 m for 30 days) were sequenced. A total of 691392 high-quality reads were produced, with an average of 57779± 6678 in NC group and 55564± 5369 in HC group (Table 2). The rarefaction curves (Fig 1) tended towards the saturation plateau while Good’s coverage estimations revealed that the amount of obtained bacterial species in NC group and HC group were 99.57%, 99.62%, respectively (Table 2), indicating that the sequencing coverage was sufficient to capture the diversity of the bacterial communities in the sample. OTU abundance, estimators of the community richness (Chao and ACE) and the diversity (Shannon and Simpson) were summarized. No significant difference was observed in OTUs, diversity index (Shannon and Simpson) and richness index (Chao and ACE) between the two groups.

**Table 2:** Summary of HiSeq sequencing data.

Sample name	Reads	OTUs	Simpson	Shannon	Chao	ACE	Goods coverage
HC	55564± 5369	940±108	0.97±0.01	6.79± 0.40	1048±118	1080±125	0.9962±0.0008
NC	57779± 6678	1138±197	0.97±0.02	7.27±0.60	1258±211	1276±190	0.9957±0.0005

Values are the mean ± standard deviation of six replicates. The number of reads, OTUs, richness estimator (Chao and ACE) and diversity estimator (Shannon and Simpson) were calculated at the 97% similarity level. NC = Mice raised in low altitude (2200 m above sea level); HC = raised in high altitude (5000 m above sea level).

### Beta diversity of gut microbiota between the two groups with multivariate statistics analysis

**Fig 2:**
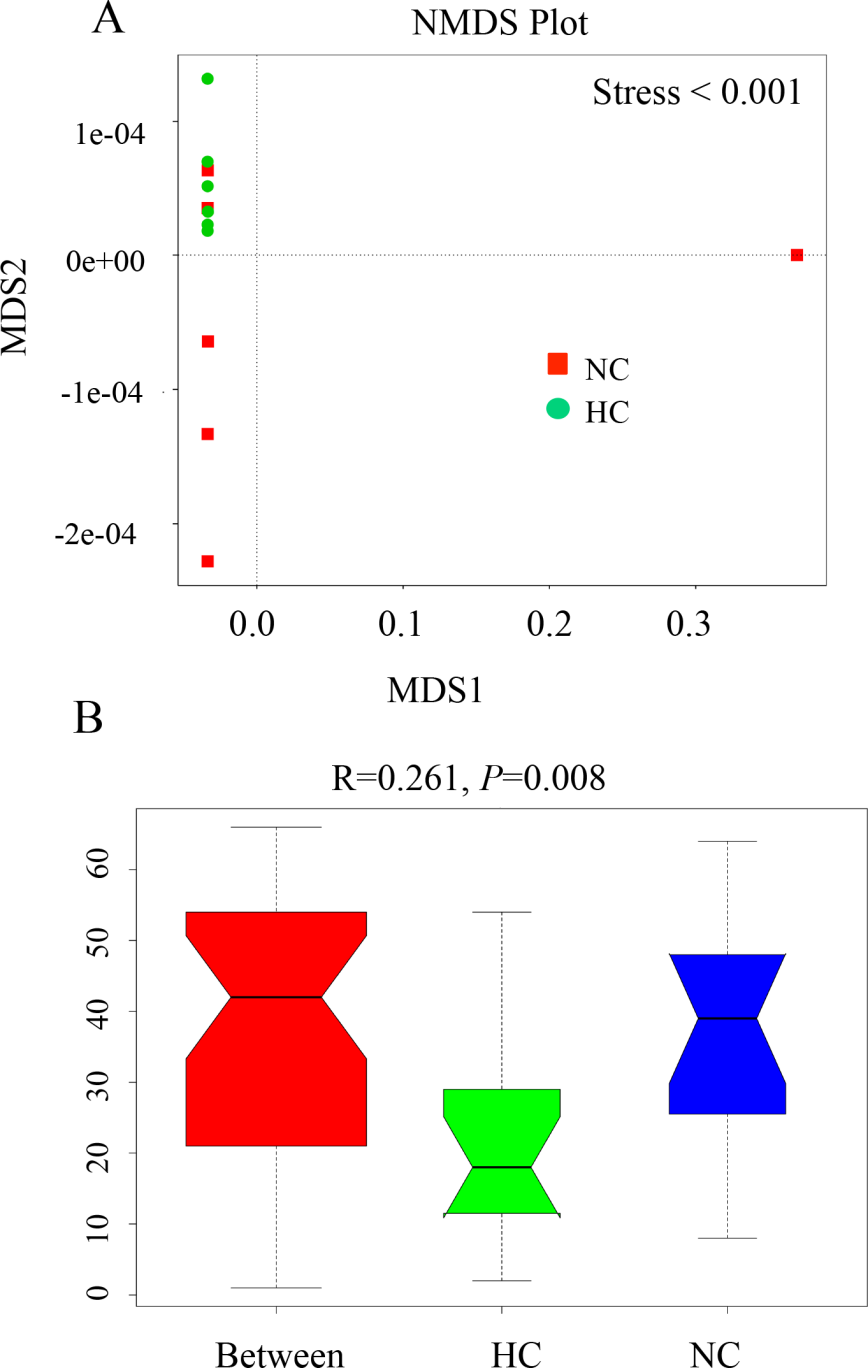
**Non-Metric Multi-Dimensional Scaling (NMDS) plot (A) and ANOSIM analysis (B).** NMDS is a simple method for visual interpretations to compare the overall structure of fecal microbiota between two samples while ANOSIM is used to statistically test the significant difference between groups.

We used Non-Metric Multi-Dimensional Scaling (NMDS) as a simple method of visual interpretations to compare the overall structure of fecal microbiota between two samples (Fig 2). NMDS was performed using the Bray-Curtis similarity index based on the relative abundance of OTUs (at a 97% similarity level). Moreover, we also described the use of analysis of similarity (Anosim) to statistically test the significant difference between groups (Fig 2). Analysis of similarity (ANOSIM) revealed that significant different were observed between NC group and HC group in microbiota community structure (R =  0.2611; *P*  = 0.008).

### Fecal microbiota composition between two groups

**Fig 3:**
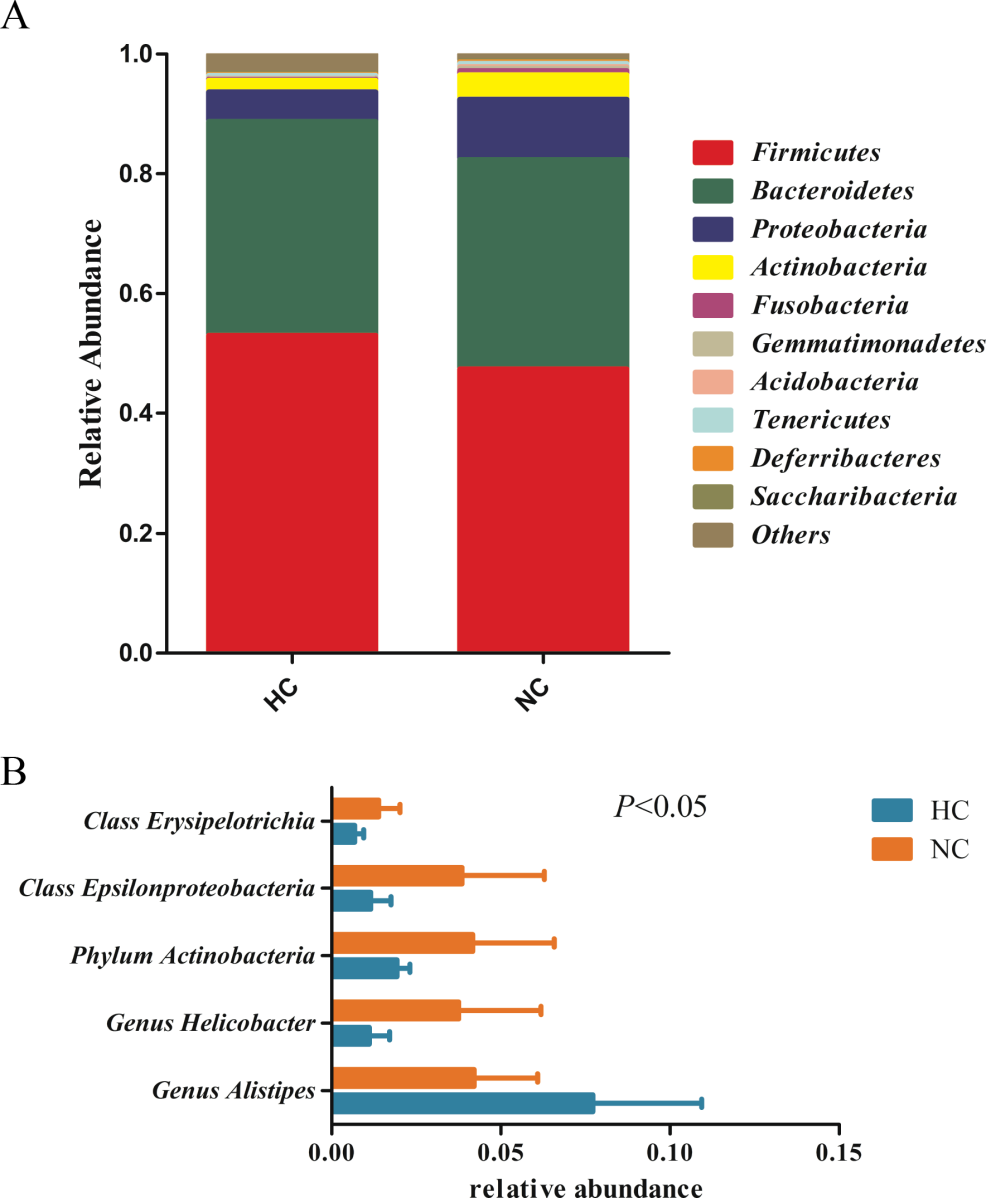
**Relative abundance of bacterial phyla (A) in fecal (Top 10), and statistical significance between two groups at phylum, class and genus levels (B).** Phylogenetic groups accounting for unclassified sequences are summarized in the artificial group ‘others’. Statistical differences between two groups were tested using t-test. Differences were considered significant at P < 0.05.

Overall bacteriome composition for each group of mice at the taxonomy level was summarized in Fig 3 and the raw data from QIIME was provided as supporting information (S1 File). The dominant phyla of these two groups of mice were *Firmicutes*, *Bacteroidetes*, and *Proteobacteria*. Compared with the NC group, the relative abundance of class *Epsilonproteobacteria*, phlym *Actinobacteria* and class *Erysipelotrichia* was significantly decreased (*P*<0.05) in HC group. At the genus level, the relative abundance of *Helicobacter* was significantly decreased (*P*<0.05), while the relative abundance of genus *Alistipes* was increased (*P*<0.05) in HC group (Fig 3).

### Phenotypic analysis

**Fig 4:**
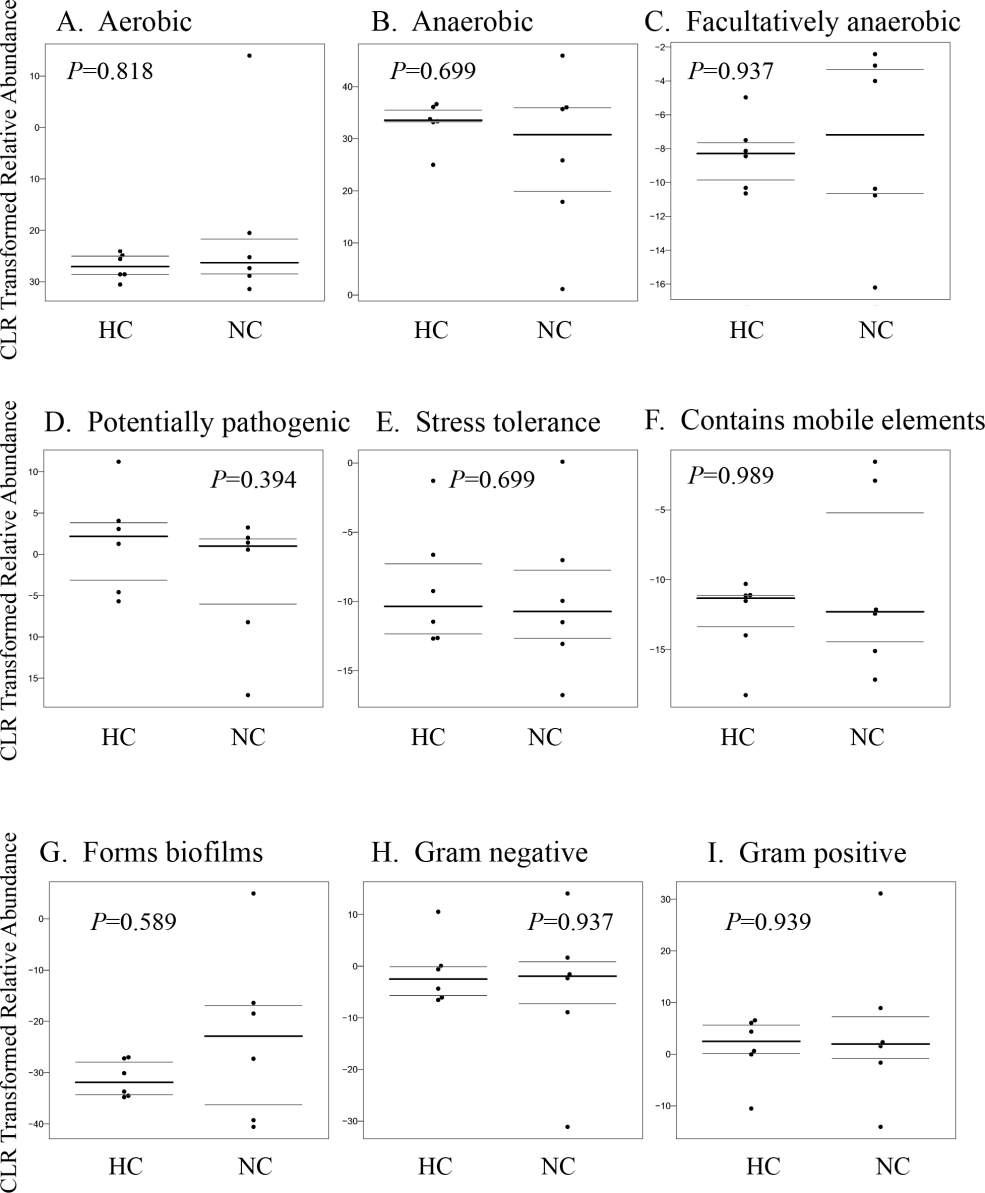
Phenotypic prediction based on BugBase analysis. BugBase is a bioinformatics tool that infers community-wide phenotypes, and predict phenotypic differences from 16S rRNA sequence data. BugBase identified that phenotypes associated with aerobic, anaerobic, facultatively anaerobic, potentially pathogenic, stress tolerance, mobile element, biofilms formation, gram negative bacteria and gram positive bacteria.

We utilized BugBase, a bioinformatics tool that infers community-wide phenotypes, predicted phenotypic differences from 16S rRNA sequence data (S2 File). BugBase identified that phenotypes associated with aerobic, anaerobic, facultatively anaerobic, potentially pathogenic, stress tolerance, mobile element, biofilms formation, gram negative bacteria and gram positive bacteria were not affected between two groups (Fig 4).

### Functional analysis

**Fig 5:**
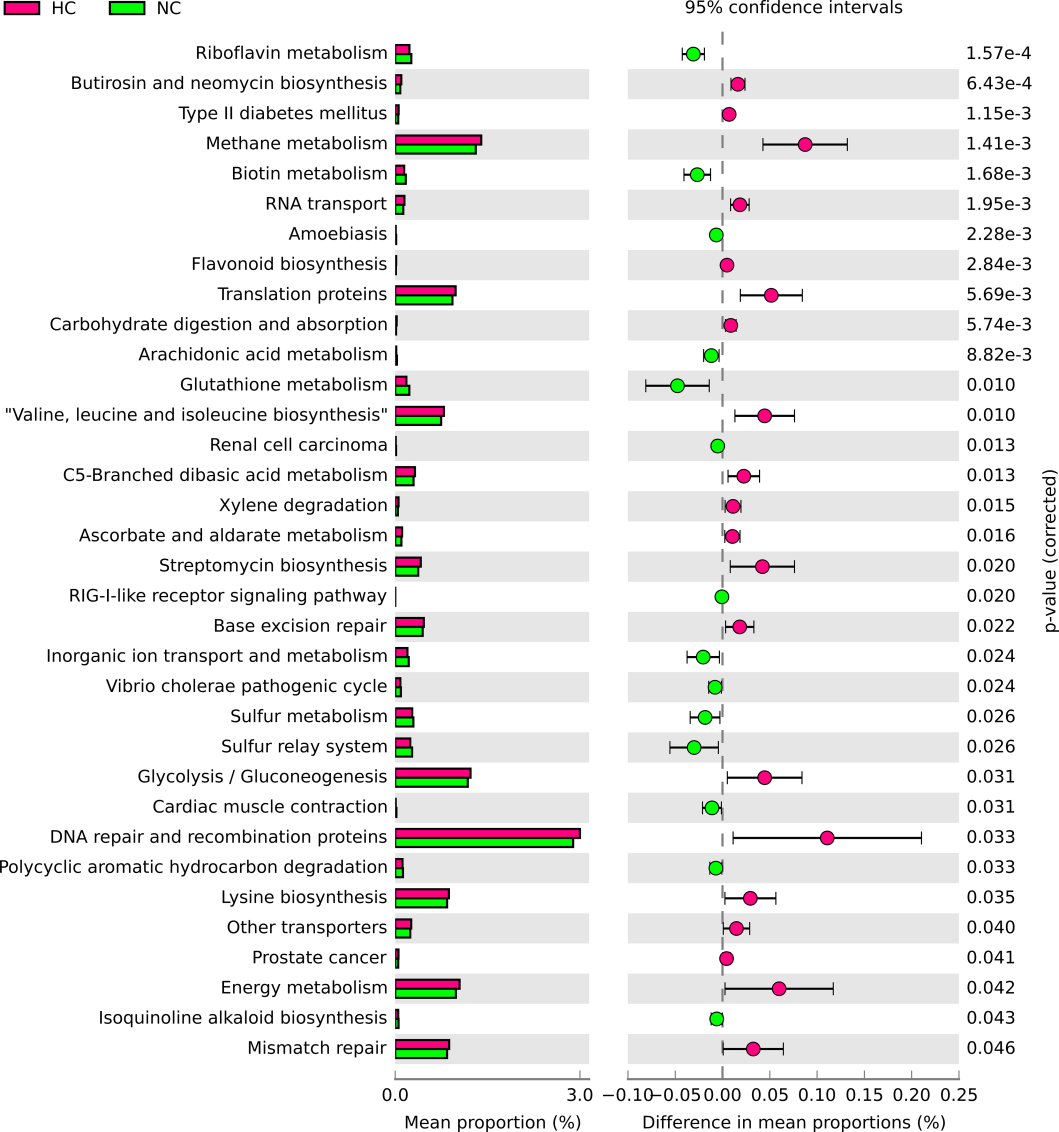
Bacterial gene functions were predicted from 16S rRNA gene-based microbial compositions using the PICRUSt algorithm to make inferences from KEGG annotated databases. Data from PICRUSt were imported into the statistical analysis of metagenomic profiles (STAMP) (version 2.1.3) package for statistical analysis and visualization. Differences were considered significant at *P* < 0.05 using t-test.

Bacterial gene functions were predicted from 16S rRNA gene-based microbial compositions using the PICRUSt algorithm to make inferences from KEGG annotated databases. The results from the PICRUSt are provided as supporting information (S3 File), and then imported into the statistical analysis of metagenomic profiles (STAMP) (version 2.1.3) package for further statistical analysis and visualization. Significant differences was observed in 34 KEGG pathways between HC group and NC group (Fig 5), such as carbohydrate digestion and absorption, energy metabolism, DNA repair and recombination proteins, RNA transport, RRIG-I-like receptor signaling pathway, flavonoid biosynthesis, translation proteins, inorganic ion transport and metabolism, etc.

## Discussion

In this current study, we determined whether the changes of geographical altitude (analogous to people from plain areas coming to the plateau for travel or work) would influence the fecal microbiota in mice. Although previous studies have shown that persons of different geographic origins may result in diverse compositions of gut microbiota, the differences might be mainly due to the distinctive life environments, genetic background, or dietary habits [11, 13]. Animal models provide the possibility of controlling these factors, potentially allowing the rational design of future human studies. We used mice as an animal model and the experimental condition was strictly controlled. In order to imitate the high-altitude hypoxic environment, we housed mice in a controlled hypobaric chamber for 30 days, and used bacterial 16S rRNA gene sequencing to compare the fecal microbial communities with the control mice that were exposed to low altitude. Overall, we found different fecal microbial communies in two groups of mice, but no statistical significant difference was observed in the alpha diversity. In line with our observations, it was shown previously that people who traveled from low-altitude areas to high altitude places (such as a 47-day exposure of the mountaineers from German to Nepalese Himalayas over 5000 meters above see level) had altered composition of intestinal microbiota [15].

In order to further investigate the effect of exposure to hypoxia at high altitude on intestinal microbiota composition, we also compared the fecal microbiota between mice raised in low altitude and mice raised in high altitude in different taxonomic levels. In genus level, we found that high-altitude hypoxia environmental exposure increased the relative abundance of genera *Alistipes*. *Alistipes* have been implied to be associated with gut inflammation and were elevated in chronic fatigue syndrome, irritable bowel syndrome and depression [25, 26]. It has been suggested that these high levels of *Alistipes* might be a potential cause for the hypoxia-induced intestinal disorders [25]. Future work should be expanded to examine the role for *Alistipes* in intestine microbial communities under high-altitude hypoxia environment. Moreover, our results provide evidence suggesting that high-altitude hypoxia environment reduces the relative abundance of genera *Helicobacter*. The majority species which belong to the genera *Helicobacter* has been proven or suspected as gastrointestinal pathogens [27]. Further work is needed to better understand the interactions between intestinal *Helicobacter* and high-altitude hypoxia. Taken together, these results suggest that hypoxia may influence the composition of the microbial community in the intestine. In line with our observations, Xu *et al*. who reported that exposure rats at hypobaric hypoxia environment for 5 days could decrease the numbers of *Lactobacillus*, *Treponema* and *Peptostreptococcaceae_Incertae_Sedis*, increase the relative number of *Prevotellaceae_uncultured* and *Prevotella* in the caecum through 454 pyrosequencing analysis [28]. Li *et al*. compared the fecal microbiota composition between Chinese Han living at the lowland and Chinese Han living in Tibet (similar diet habits and genetic background), and barcoded 454 pyrosequencing results showed that significant differences in the relative abundances of 13 genera among the two groups [13].

Interestingly, our study showed that high-altitude hypoxia environment could significantly decrease the abundance of class *Epsilonproteobacteria*, phylum *Actinobacteria* and class *Erysipelotrichia*, which were obligatory or facultatively aerobic mostly [29, 30]. However, the phenotypic analysis indicated that exposure to high altitude and low oxygen didn’t change the relative abundance of aerobic, anaerobic, facultatively anaerobic, potentially_pathogenic, gram negative bacteria and gram positive bacteria in mice. Using bacterial culture *in vitro* approach, Adak *et al*. found that total aerobes decreased significantly with the increase of total and facultative anaerobes in 15 soldiers during their 15-day acclimatization at 3,505 m high-altitude [16]; Samanta *et al*. reported that the count of total aerobes and facultative anaerobes decreased while those for total anaerobes increased in rats exposed to hypobaric pressure for 7 days[31]. The differences between these reported observations and our results may be related with the methodology used in different studies. For example, we focused on the whole microbial communities rather than culturable microbiota.

The inference of microbial function using PICRUSt added another dimension in characterizing microbiota difference between mice raised in high altitude and those raised in low altitude. It is well known that high altitude hypoxia exposure causes changes in physiological and metabolic processes due to hypobaric hypoxia [32, 33]. Functional analysis demonstrated microbial communities influenced by high altitude hypoxia were related to several metabolism pathways, which further indicated that hypoxia at high altitudes could modify microbial communities and subsequently cause physiological and metabolic dysfunction. The relative abundance of genes involved in the carbohydrate digestion and absorption, and energy metabolism were increased in a high altitude hypoxia environment. It has been reported that a disturbed energy balance was caused by a reduction of intake, an increased requirement due to the acute mountain sickness [34]. We hypothesized that intestine microbiota helped meet the body’s energy requirements through expediting carbohydrate digestion and absorption. Functional pathways, which were depleted, might be associated with the potential health consequences. For instance, our analysis revealed genes involved arachidonic acid metabolites, glutathione metabolism, inorganic ion transport and metabolism, RIG-I-like receptor signaling pathway, DNA repair and recombination protein, sulfur metabolism and sulfur relay system were decreased under high altitude hypoxia environment. Glutathione, acting as a co-factor in the synthesis of proteins and deoxyribonucleotides, protected against cellular damage due to reactive oxygen species and toxic chemicals of both endogenous and xenobiotic origin. Decreased glutathione content was found in the livers and erythrocytes of hypoxia-exposed rats, which might explain the increased production of reactive oxygen species (ROS) and impairment of xenobiotic metabolism during exposure to high altitude hypoxia [32]. Interaction with various ion transport proteins was one of ROS’s deleterious effects [35], which might be the cause of decreased genes involved with inorganic ion transport and metabolism. Arachidonic acid metabolites are responsible for hypoxia-induced headache [36]. Similar result was observed that shift in production of arachidonic acid metabolites occurs during exposure to short hypoxia in weaned pigs [37]. Decreased genes involved with sulfur metabolism and sulfur relay system might be associated with decreased *Epsilonproteobacteria*. *Epsilonproteobacteria* is in general associated with sulfide rich environments where they play a key role in the cycling of carbon, nitrogen, and sulfur [38]. Moreover, RIG-I-like receptors, known as cytoplasmic sensors of pathogen-associated molecular patterns (PAMPs) within viral RNA, play a major role in sensing RNA virus infection to initiate and modulate antiviral immunity [39]. Gene-family expansion in genes associated with DNA repair was enriched in Tibetan antelope which has lived at high altitude for a long time [40]. However, other functional pathways (biotin metabolism, etc) were not observed in other studies. It will require further confirmation to better characterize the functional effects of altitude induced microbiota compositional changes.

In summary, our results suggested that exposure of low oxygen and high altitude had the potential to influence intestinal microbial composition.

## Supporting information

S1 File Fecal microbiota composition between two groups. (XLS)

S2 File Phenotypic prediction based on BugBase analysis. (TXT)

S3 File Bacterial gene functions were predicted from 16S rRNA gene-based microbial compositions using the PICRUSt algorithm to make inferences from KEGG annotated databases. (TXT)
